# Supplementary material for: Use of instructional videos in leadership education in higher education under COVID-19: A qualitative study
Source: PLoS One. 2023 Sep 21;18(9):e0291861. doi: 10.1371/journal.pone.0291861 (PMC10513278; doi:10.1371/journal.pone.0291861)
Supplement: S1 Dataset — (DOCX) [file pone.0291861.s001.docx]

**Minimal Data Set**

Manuscript title: Use of Instructional Videos in Leadership Education in Higher Education under COVID-19: A Qualitative Study

Table 1. First and second order codes on the use of videos by students and teachers and arrangement of videos

| **Theme** | **Second Order Codes** | **First Order Codes** |
| --- | --- | --- |
| Theme 1: Video Arrangement | Variation in students’ use of videos | - Frequency of video watching (e.g., one time, watched the videos again if encountered difficulties) - Time spent (e.g., 10 to 20 minutes, around 30 minutes, half an hour to one hour) - Viewing time (e.g., watched before class) - Purpose of watching (e.g., watched the videos again for learning or assignment, revision materials or shortcut to the course content) |
|  | Variation in teachers’ use of videos | - Some did not play the videos again during lecture - Some utilized the videos (e.g., recapped and explained) - Played or recapped the videos again benefited students - Drawbacks of playing or recapping the videos during lecture (e.g., might be boring for students who had watched the videos) |
|  | Positive effect of video arrangement | - Flexible learning - Low demand - Increased students’ concentration, understanding and engagement - Reduction in class duration enhanced students’ concentration in lecture - Video watching counted towards students’ participation grade |
|  | Close relationship between the theory videos and the lectures | - Lecturer discussed or recapped the concepts in the theory videos during lecture - Video-related class activities could facilitate students’ learning - Videos and lecture topics were related - Videos enabled students to concentrate better in lecture - Lecturers discussed case scenario with students during lecture - Lecturer used personal experience or real life examples to explain the theories - Ways the theory videos and the lectures were not closely related (e.g., some theories were not explained clearly in lecture) |
|  | Close relationship between the case-based videos and the lectures | - Case-based videos and the lectures are closely related (e.g., lecturer explained the concepts again in lecture, thought-provoking, lecturers would share personal experience in lecture) - The case-based videos and the lectures are not closely related (e.g., lecturers would share new cases or personal experience rather than the case-based videos in lecture) |
|  | Drawback of video arrangement | - Watch the videos before lectures has drawbacks (e.g., not able to ask lecturer questions immediately after watching the videos) - Workload was too big - Workload beyond expectations |
|  | Suggestions on video arrangement | - Suggestions on video arrangement (e.g., preferred face-to-face lecture) - Difficulties encountered (e.g., not able to access the files anymore after the lecture) - Ways to improve students’ understanding of the theories (e.g., more interactive activities) - Ways to improve the videos (e.g., combining all theory videos into one long video) - Ways to enhance the benefit to students’ learning (e.g., reducing the number of blended learning questions before lecture) |

Table 2. First and second order codes on video design

| **Theme** | **Second Order Codes** | **First Order Codes** |
| --- | --- | --- |
| Theme 2: Video Design | Positive aspects | - Good video design (e.g. role play) - Good combination of theory video and case-based video |
|  | Negative aspects and ways of improvement | - Problems encountered (e.g., audio problems) - Ways to improve the quality of the videos (e.g., reducing the video length, improve audio quality) |
|  | Various features of the theory videos contributed to students’ learning | - Students enjoyed the theory videos - Lecture time could be reserved for lecturer sharing personal experiences or class discussion. - Features of the theory videos that benefited students’ learning (e.g., very structured and explained the theories clearly, model explanation) - The blended learning questions enhanced understanding - The examples and questions helped students to develop critical thinking skills - Visual and audio stimulation improved concentration - Discussion or interactive mode for theory learning was interesting |
|  | Different features of case-based videos benefited students’ learning | - Students enjoyed the case-based videos (e.g., related to students’ lives) - Facilitating features (e.g., helped students relate theory to the case, students could relate the cases to social issue) - Features not benefiting students’ learning (e.g., some videos did not provide solutions and did not analyze different factors) |

Table 3. First and second order codes on video content

| **Theme** | **Second Order Codes** | **First Order Codes** |
| --- | --- | --- |
| Theme 3: Video Content | Suggestions for improvement on video content | - Difficulties encountered (e.g., some theory videos are boring) - Ways to improve video quality (e.g., adding translation of vocabulary in the subtitle, enhancing the link between the blended learning questions and the videos, add examples or news) - Ways to strengthen students’ understanding (e.g., discuss more social issues in the video) |
|  | Connection between theory videos and case-based videos | - The majority of students thought that the theory videos are related to the case-based videos (e.g., the videos talked about the theory first, then presented the cases) - Ways the theory videos are related to the case-based videos (e.g., the case could explain the theory, students could apply theories into the case) - A few negative views: The theory videos are not strongly related to the case-based videos. |
|  | Suggestions for improvement on connection between theory videos and case-based videos | - Ways to strengthen the links between the theory videos and the case-based videos (e.g., add more real life application, more variety of video format, simplify the videos) |

Table 4. First and second order codes on videos’ benefits to the students’ pre-lesson self-learning

| **Theme** | **Second Order Codes** | **First Order Codes** |
| --- | --- | --- |
| Theme 4: Videos’ Benefits to the Students’ Pre-lesson Self-learning | The theory videos promote self-learning | - Promote student understanding (e.g., clear explanation of theories) - Effective learning tool - Facilitates term paper writing - Provided an overall mind map or guidelines - Explains concepts via diagrams - Problems hindering self-learning (e.g., some English vocabularies were difficult to understand, videos were too long) |
|  | The case-based videos promote self-learning | - Videos are clear enough for self-learning (e.g., suitable for pre-lecture preparation, helped students’ term paper writing) - Videos are not suitable for self-learning (e.g., easy to understand but may not motivate students to reflect) |

Table 5. First and second order codes on videos’ benefits to the students’ learning of course content

| **Theme** | **Second Order Codes** | **First Order Codes** |
| --- | --- | --- |
| Theme 5: Videos’ Benefits to the Students’ Learning of Course Content | The videos’ general benefits to students’ learning | - Advantages of the videos (e.g., reduced lecture time enhanced concentration) - Learning with self-discipline and learn to manage time - Benefits to academic study - Self-pace their learning - Promote understanding of lecture content (e.g., videos serve as class preparation material) - Promote understanding of theories related to the topic - Ways the videos enhanced students’ understanding of theories (e.g., enhance students’ self-reflection) - High satisfaction with the videos |
|  | Examples on how the theory videos benefited students’ learning | - Explained the theories clearly, which helped students to understand without prior knowledge - The topics were related to students’ daily lives - Could stimulate students’ self-reflection - The animation, layout and video cutting interesting - Concrete examples explained the theory and illustrated the strategies used - The lecturer shared students’ blended learning answers during lecture, which were interesting to students |
|  | Suggestions for improving the theory videos | - Adding more cases or examples to explain the theory and definitions - Rethinking how lecture could be linked to the theory videos - Ways the theory videos’ could not benefit student learning (e.g., too many concepts and the focuses of the theories were not clear, the narration of the theory videos directly read out the definition, which was boring) |
|  | The case-based videos benefited students’ learning | - Case-based videos impressed students through different means (e.g., related to students’ daily lives and the social issue, gained more positive mindset) - Students could use the strategies in their daily lives - Related to the social issue, facilitate students to understand the theory very easily - Entertaining and surprising, so students would keep on thinking about the videos after the lecture - The cartoon was interesting - Role-play videos were engaging |
|  | Suggestions for improving the case-based videos | - The course should be taught through face-to-face lecture rather than online lecture or videos. Human interaction is important for their learning. - Case-based videos with real actors - Adding more small cases after each theory - Have more interaction with the lecturers - Ways the case-based videos could not benefit student learning (e.g., some cases were not real or not related to students’ lives so students could not relate, no room for discussion) |

Table 6. First and second order codes on videos’ benefits to the students’ class participation

| **Theme** | **Second Order Codes** | **First Order Codes** |
| --- | --- | --- |
| Theme 6: Videos’ Benefits to the Students’ Class Participation | Enhancement of class participation | - Could be used for class discussion, which increased students’ motivation - Had time to understand and think about the topic before lecture - How the videos promoted students’ participation during class (e.g., able to answer lecturers’ questions, gained more interest after knowing the course content from the videos, had flexibility to control learning pace) - Reasons why videos did not affect students’ class participation (e.g., class participation depended on lecturers’ leadership) - Reasons the videos may hinder students’ class participation (e.g., already knew the course content from the videos, so students were less concentrated in class discussion) |
