# Supplementary material for: Use of instructional videos in leadership education in higher education under COVID-19: A qualitative study
Source: PLoS One. 2023 Sep 21;18(9):e0291861. doi: 10.1371/journal.pone.0291861 (PMC10513278; doi:10.1371/journal.pone.0291861)
Supplement: Attachment — Submitted filename: Response to Reviewers_20230819.docx [file pone.0291861.s002.docx]

Dear Editor,

Manuscript title: Use of Instructional Videos in Leadership Education in Higher Education under COVID-19: A Qualitative Study

I would like to express my sincere gratitude to the reviewers’ comments. Below are our point-by-point response to the comments.

1. “Although authors mentioned triangulation at the end of the study, reliability and validity of measurements and results should be reported in detail including demographics of participants.”

Response: Thank you very much for your comment. Validity and reliability are important concepts in qualitative research to ensure trustworthiness of the findings and rigor of the research. In this study, different strategies were integrated in the research design and methodology to ensure the validity and reliability of the findings. First, all the focus group interviews were audio-recorded, which allowed researchers to check the truthfulness of the emerging themes (Noble & Smith, 2015). Second, rich verbatim transcription extracts were provided in the manuscript, which enabled readers to judge the truthfulness of the final themes themselves (Creswell & Miller, 2000; Denzin, 1989). These two strategies ensured the findings represented the phenomena, thus enhancing the research validity. Third, emerging themes were discussed transparently among researchers in the project, which minimized biases in the research method (Noble & Smith, 2015; Lincoln & Guba, 1985). Fourth, inter-rater reliability test was conducted and the results showed that the two coders agreed on 18 out of 20 codes. This showed a high level of inter-rater reliability in the analyses (Huberman & Miles, 1994; Shek et al., 2005). These two strategies ensured that researchers arrived at similar codes or themes in the data analysis, thus enhancing the reliability of the current research. Lastly, triangulation of the findings in the present study and our previous quantitative research findings was conducted to enhance the research validity (Creswell & Clark, 2011; Creswell & Miller, 2000; Golafshani, 2003). Our previous quantitative research was conducted on the same cohort of students to assess their satisfaction with and benefit received from watching the same batch of videos under the blended learning and flipped classroom arrangement. The side-by-side comparison of the findings showed that results from the current qualitative study and previous quantitative study aligned with each other. This showed that the current research findings could accurately present research participants’ perspective, thus demonstrating the validity of the current research. Supporting literature was added to the citation and reference list of the manuscript.

Analysing the demographics of research participants is important to understand whether findings of the current research could transfer to other settings or contexts (Agius, 2013; Noble & Smith, 2015). The current research did not collect demographic information from the students. Studies suggested that maintaining research participants’ anonymity could encourage them to disclose more opinion during the interview (Joinson, 2001; Opdenakker, 2006). The sampled participants were all year 1 students in the case university. The general student demographic information in the case university showed that the mean age of year 1 students in the university is 18 and there is an equal ratio of male to female students (The Hong Kong Polytechnic University, 2022). These student demographic characteristics were generally similar to other Chinese universities (Long et al., 2016) and international universities (Browning et al., 2021). Therefore, the findings of the current study could be generalized to students in Chinese or international contexts. However, it is important to note that students’ perceived benefits from watching educational videos were influenced by various factors, including the subject taught and external social factors (e.g., COVID-19 pandemic; Joko Prayudha, 2021; Lacey & Wall, 2021; Mao et al., 2022). Moreover, researchers also noted that students’ financial status could affect their ability to acquire electronic devices, thus influencing the effectiveness of electronic learning (Alsoufi et al., 2020). Readers should consider these factors when applying the current research findings to other contexts. Supporting literature was added to the citation and reference list of the manuscript.

2. “In addition, more practical applications and future research suggestions should be added at the end of the manuscript.”

Response: Thank you very much for your comment. Practical applications and future research suggestions were added in the discussion section. Supporting literature was added to the citation and reference list of the manuscript. For practical applications, this study demonstrated the teaching and learning effectiveness of 24 educational videos under a blended learning and flipped classroom arrangement. Education practitioners could consider adopting similar practice in the future, particularly in the online teaching and learning context. These educational videos are very valuable under the pandemic, and they could be further used in other situations that require online education (e.g., courses for students in remote areas). Moreover, this study showed that various video design features could enhance university students’ learning effectiveness, which not only supported the multimedia learning theories, but also provided design reference for education practitioners when developing videos for university students.

For future research suggestions, researchers were recommended to further investigate how different video arrangement and video design features could benefit students’ learning at higher education level. Besides, more qualitative research or mixed method studies could be implemented to evaluate the effectiveness of educational videos in different contexts. This could accumulate evidence on how educational videos benefits students’ learning and contribute to evidence-based teaching practice.

3. “One point which I feel it is important is that self tests which I think should be given post lecture and the students should have the opportunity to repeat the tests multiple times in order to score the highest grade possible, and such evaluations for students should be in an interactive form of tests.”

Response: Thank you very much for your comment. Although the majority of students in the current study agreed that they were able to gain more understanding and participate more in class discussion under the flipped classroom arrangement, some students also found that it was stressful for them to understand so many concepts and to complete the blended learning exercises before attending lectures. Therefore, some of them preferred watching the videos and completing the blended learning exercises after lectures, so they would have better understanding of the topics after teachers’ explanation in lectures. We have added more analysis of pre- and post-lecture learning activities in the discussion section. Supporting literature was added to the citation and reference list of the manuscript.

Sincerely Yours,

Daniel T.L. Shek, Tingyin Wong, Xiang Li, Lu Yu

**References**

Agius, S. J. (2013). Qualitative research: Its value and applicability. *The Psychiatrist, 37*, 204-206. <https://doi.org/10.1192/pb.bp.113.042770>

Alsoufi, A., Alsuyihili, A., Msherghi, A., Elhadi, A., Atiyah, H., Ashini, A., Ashwieb, A., Ghula, M., Hasan, H. B., Abudabuos, S., Alameen, H., Abokhdhir, T., Anaiba, M., Nagib, T., Shuwayyah, A., Benothman, R., Arrefae, G., Alkhwayildi, A., Alhadi, A., ... & Elhadi, M. (2020). Impact of the COVID-19 pandemic on medical education: Medical students’ knowledge, attitudes, and practices regarding electronic learning. *PloS one, 15*(11):e0242905. <https://doi.org/10.1371/journal.pone.0242905>

Browning, M. H., Larson, L. R., Sharaievska, I., Rigolon, A., McAnirlin, O., Mullenbach, L., Cloutier, S., Vu, T. M., Thomsen, J., Reigner, N., Metcalf, E. C., D'Antonio, A., Helbich, M., Bratman, G. N., & Alvarez, H. O. (2021). Psychological impacts from COVID-19 among university students: Risk factors across seven states in the United States. *PloS one, 16*(1):e0245327. <https://doi.org/10.1371/journal.pone.0245327>

Creswell, J. W., & Clark, V. P. (2011). *Mixed Methods Research* (2^nd^ ed.). Thousand Oaks, CA: Sage.

Creswell, J. W., & Miller, D. L. (2000). Determining validity in qualitative inquiry. *Theory into Practice, 39*(3), 124-130. <https://doi.org/10.1207/s15430421tip3903_2>

Denzin, N. K. (1989). *Interpretive Interactionism.* Newbury Park, CA: Sage.

Golafshani, N. (2003). Understanding reliability and validity in qualitative research. *The Qualitative Report, 8*(4), 597-607.

Huberman, A. M., & Miles, M. B. (1994). *Qualitative Data Analysis: An Expanded Sourcebook*. Thousand Oaks, CA: Sage.

Joko Prayudha, S. (2021). Video based learning as a media for teaching English during pandemic covid-19. *Journal of Language Intelligence and Culture, 2*(1), 1-11. <https://doi.org/10.35719/jlic.v2i1.53>

Joinson, A. N. (2001). Self-disclosure in computer-mediated communication: The role of selfawareness and visual anonymity. *European Journal of Social Psychology, 31*, 177-192.

Lacey, K., & Wall, J. G. (2021). Video-based learning to enhance teaching of practical microbiology. *FEMS Microbiology Letters, 368*(2):fnaa203. <https://doi.org/10.1093/femsle/fnaa203>

Lincoln, Y. S., & Guba, E. G. (1985). *Naturalistic Inquiry*. Beverly Hills, CA: Sage.

Long, J., Liu, T. Q., Liao, Y. H., Qi, C., He, H. Y., Chen, S. B., & Billieux, J. (2016). Prevalence and correlates of problematic smartphone use in a large random sample of Chinese undergraduates. *BMC Psychiatry, 16*:408. <https://doi.org/10.1186/s12888-016-1083-3>

Mao, B. P., Teichroeb, M. L., Lee, T., Wong, G., Pang, T., & Pleass, H. (2022). Is online video-based education an effective method to teach basic surgical skills to students and surgical trainees? A systematic review and meta-analysis. *Journal of Surgical Education, 79*(6), 1536-1545. <https://doi.org/10.1016/j.jsurg.2022.07.016>

Noble, H., & Smith, J. (2015). Issues of validity and reliability in qualitative research. *Evidence-based Nursing, 18*(2), 34-35. <https://doi.org/10.1136/eb-2015-102054>

Opdenakker, R. J. G. (2006). Advantages and disadvantages of four interview techniques in qualitative research. *Forum Qualitative Sozialforschung = Forum: Qualitative Social Research, 7*(4):Art. 11.

Shek, D. T., Tang, V. M., & Han, X. Y. (2005). Evaluation of evaluation studies using qualitative research methods in the social work literature (1990-2003): Evidence that constitutes a wake-up call. *Research on Social Work Practice, 15*(3), 180-194. <https://doi.org/10.1177/1049731504271603>

The Hong Kong Polytechnic University. (2022). *Facts and Figures*. Institutional Planning and Analytics Office. <https://www.polyu.edu.hk/en/ipao/facts-and-figures/>
